# Essential roles of CTCF binding sites at TAD boundaries in modulating chromatin interactions and transcriptional regulation at the *Ifng* locus

**DOI:** 10.3389/fimmu.2025.1667851

**Published:** 2025-09-26

**Authors:** Kazuya Muto, Daiki Yoshida, Toshiyuki Suzuki, Natsuo Yamamoto, Naokazu Inoue, Akiko Murakami-Sekimata, Ken Iseki, Masayuki Sekimata

**Affiliations:** ^1^ Radioisotope Research Center, Fukushima Medical University School of Medicine, Fukushima, Japan; ^2^ Department of Emergency and Critical Care Medicine, Fukushima Medical University School of Medicine, Fukushima, Japan; ^3^ Sendai City Public Health Center, Miyagi, Japan; ^4^ Department of Cell Science, Institute of Biomedical Sciences, Fukushima Medical University School of Medicine, Fukushima, Japan; ^5^ Department of Life Sciences and Nursing, Fukushima Medical University School of Nursing, Fukushima, Japan

**Keywords:** IFN-g, CTCF, TAD, intra-TAD, enhancer, transcription, chromatin architecture

## Abstract

Interferon-γ (IFN-γ) is a key cytokine essential for host defense and tumor surveillance, and its expression in T cells is tightly regulated by long-range enhancer–promoter interactions within the *Ifng* locus. These interactions are organized within topologically associating domains (TADs) and subdomains (intra-TADs), whose boundaries are defined by the architectural protein CTCF. However, the specific contribution of these boundaries to *Ifng* regulation and chromatin architecture remains incompletely understood. Here, we investigated how chromatin looping within the TAD affects *Ifng* expression by using chromosome conformation capture (3C) and RNA sequencing in CRISPR-Cas9 genome editing in mice. Deletion of a single CTCF binding site (CBS) at the TAD boundary markedly impaired Th1-mediated immune responses against *Cryptococcus neoformans* infection and B16 melanoma. This deletion disrupted enhancer–promoter contacts and diminished enhancer-driven activation of *Ifng*. In contrast, deletion of a CBS within an intra-TAD boundary had no detectable impact on *Ifng* transcription. Collectively, these findings highlight the essential role of boundary-associated CBS elements in shaping chromatin architecture that enables enhancer–promoter communication and proper transcriptional regulation of the *Ifng* locus.

## Introduction

Gene expression is a highly complex and tightly regulated process that is fundamental to establishing cell identity and maintaining organismal homeostasis. This intricate regulation is especially crucial during immune responses, which require precise spatiotemporal coordination of transcriptional programs in response to diverse antigens and microenvironmental cues. Cytokines, the key mediators of immune responses, are subject to strict control by multiple *cis*-regulatory elements, including promoters, enhancers, silencers, and insulators, often located within their genomic loci (1, 2). Although the individual roles of these elements have been extensively characterized, increasing evidence suggests that their dynamic long-range interactions within the nucleus represent a crucial third layer of regulation essential for robust cytokine gene expression. CD4^+^ T helper (Th) cells offer an ideal model for examining such chromatin interactions, owing to their accessibility and ability to differentiate into distinct functional subsets.

Th cell subsets are defined by their signature cytokine profiles and play essential roles in host defense and tumor surveillance (3, 4). Th1 cells, in particular, are characterized by their production of Interferon-γ (IFN-γ), a key effector cytokine required for clearing intracellular pathogens such as viruses and certain bacteria (5). In contrast, Th2 cells produce low levels of IFN-γ and instead secrete type 2 cytokines (e.g., IL-4, IL-5, IL-13), which are associated with tissue inflammation and allergic responses (6). The differentiation of Th1 cells from naive CD4^+^ T cells is tightly regulated by the lineage-defining transcription factors such as T-bet, along with STAT4 and NFAT, which act in coordination with histone-modifying and chromatin-remodeling enzymes (7). T-bet not only activates the *Ifng* promoter directly but also binds to multiple conserved noncoding sequences (CNSs) across the *Ifng* locus. The identification of transcription factor binding at these CNSs (8–10) has enabled functional dissection of key enhancers, including CNS–54, CNS–34, CNS–22, CNS–6, CNS + 18/20, and CNS + 29, all of which are critical for proper *Ifng* expression (9–12). Functional evidence for their enhancer activity has been provided by reporter assays (9–12), and their roles are further supported by enrichment of the active histone mark H3K27ac at these loci (13). Notably, chromosome conformation capture (3C) studies have demonstrated T-bet-dependent chromatin loop formation between these CNSs and the *Ifng* promoter (14, 15), suggesting that these *cis*-regulatory elements function through chromatin looping mechanisms.

In addition to these positive regulatory elements, a recent study identified a silencer element (CNS–28) within the *Ifng* locus that restrains *Ifng* expression in a GATA3-dependent but T-bet-independent manner (16). This silencer functions by reducing enhancer–promoter interactions and ensures immune cell quiescence, thereby preventing inappropriate IFN-γ activation and minimizing the risk of autoimmunity. The discovery of CNS–28 highlights that both activating and repressive *cis*-elements cooperate to fine-tune *Ifng* regulation, adding another layer of complexity to chromatin-based control of cytokine transcription.

The role of three-dimensional (3D) genome architecture in gene regulation has become a focus of intense research. Recent advances in 3C-based technologies have revealed that the mammalian genome is hierarchically organized into topologically associating domains (TADs) and subdomains (intra-TADs or sub-TADs) (17–20). While TADs tend to be conserved across cell types, intra-TADs are more dynamic and can undergo structural changes during differentiation (21, 22). TADs often contain multiple intra-TADs and serve as structural frameworks that facilitate enhancer–promoter proximity and interactions (23). TAD and intra-TAD boundaries are enriched for CCCTC-binding factor (CTCF), which binds DNA at divergent motif orientations and mediates cohesin retention through its N-terminal domain to drive loop extrusion (23–26). These structural domains have been shown to play essential roles in regulating enhancer–promoter interactions in several gene clusters, such as the protocadherin and α-globin loci (22, 27). Disruption of CTCF binding sites (CBSs) can lead to aberrant chromatin topology, dysregulated gene expression, and disease phenotypes (28, 29). At the *Ifng* locus, our previous work showed that CTCF facilitates chromatin looping between CBSs and T-bet-bound enhancers, contributing to *Ifng* gene regulation (15). More recently, it was reported that deletion of a CBS at the *Ifng* locus alters local chromatin architecture and dysregulates *Ifng* expression, resulting in increased pathogen susceptibility (13). These observations underscore the *Ifng* locus as a powerful model system to study how TAD and intra-TAD structures regulate gene expression in immune cells. However, the precise mechanisms by which multiple CBSs contribute to chromatin topology and transcriptional regulation at this locus remain incompletely defined.

In this study, we provide direct evidence that CTCF-mediated chromatin looping is critical for establishing and maintaining proper enhancer–promotor communication and immune function at the *Ifng* locus. Using CRISPR-Cas9 genome editing, we individually deleted two murine CBSs located at distinct architectural positions. We found that deletion of a convergent CBS at a TAD boundary disrupted chromatin loop formation, impaired IFN-γ production, and weakened host defense against pathogens and tumors. Chromosome conformation analysis (3C and Region-Capture Micro-C) revealed that this boundary-associated CBS was essential for maintaining enhancer–promoter interactions. In contrast, deletion of a CBS within an intra-TAD region had no significant impact on *Ifng* expression. Collectively, these findings highlight the essential role of CTCF in organizing the 3D chromatin architecture at the *Ifng* locus to support transcriptional activation and effective immune responses.

## Materials and methods

### Mice

C57BL/6J, B6D2F1, and ICR mice were purchased from Japan SLC. B6(Cg)-Rag2^tm1.1Cgn^/J (Rag2^–/–^) mice were obtained from The Jackson Laboratory. *Ifng* –70kb and +66kb CTCF site mutant mice (ΔCBS–70 and ΔCBS+66 mice, respectively) were generated using CRISPR-Cas9 technology to delete the 20-bp core of their CBSs, as previously described (30). Briefly, the following pairs of single-guide RNA (sgRNA) sequences were annealed and cloned into the *Bbs*I sites of the pX330 vector (Addgene plasmid #42230): for ΔCBS–70 mice (5´-CACCGTGGAACAAATTTACCGAAGC-3´ and 5´-AAACGCTTCGGTAAATTTGTTCCAC-3´; 5´-CACCGATACCACTGCCCCACCTTGT-3´ and 5´-AAACACAAGGTGGGGCAGTGGTATC-3´), and for ΔCBS+66 mice (5´-CACCGTGCCACCTCAGGGATACAAG-3´ and 5´-AAACCTTGTATCCCTGAGGTGGCAC-3´; 5´-CACCGGTTCAGCTTCCATGTTGCT-3´ and 5´-AAACAGCAACATGGAAGCTGAACC-3´). These plasmids (5 ng/µL in 5 mM Tris-HCl [pH 7.4] containing 0.1 mM EDTA) were injected into the pronuclei of fertilized eggs from B6D2F1 mice using glass capillaries and a micromanipulator (FemotoJet, Eppendorf). Fertilized eggs were collected from B6D2F1 females, which were euthanized by cervical dislocation prior to ovary dissection. Embryos that reached the two-cell stage after overnight culture in KSOM medium (ARK Resource) were then implanted into the oviducts of pseudopregnant ICR foster mothers. For embryo transfer, pseudopregnant foster mothers were anesthetized with a mixed anesthetic consisting of medetomidine (0.3 mg/kg), midazolam (4.0 mg/kg), and butorphanol (5.0 mg/kg), administered intraperitoneally. No other anesthetics were used in this study. Offspring were genotyped by PCR amplification of the targeted regions, followed by DNA sequencing. The primers used were: 5´-GCTGCTCTGGGTCGTTTAAA-3´ and 5´-ATTTTCCACCGCAATGCCTG-3´ for ΔCBS–70 mice; 5´-CCTAACTTTCCTGACACTGATTATTGG-3´ and 5´-GGCATTGCTTGGGTAAGGTAC-3´ for ΔCBS+66 mice. The genome-edited mice with the desired deletions were backcrossed with C57BL/6J wild-type mice for more than eight generations to ensure a uniform genetic background. Mice not used for experiments were euthanized by cervical dislocation. At the end of experimental procedures, mice were also euthanized by cervical dislocation, the same method used for non-experimental animals. Surplus animals not used in experiments were euthanized to comply with animal welfare guidelines and avoid unnecessary housing. Death was confirmed by the absence of heartbeat, respiration, and reflex responses (toe pinch). For experiments, mice were age- (8–14 weeks old) and sex-matched, with littermates used as controls. Mice were maintained under specific pathogen-free conditions. All experimental procedures were approved by the Animal Experiments Committee of Fukushima Medical University and performed in accordance with our institutional regulation regarding animal experiments.

### Cell culture

All primary cells isolated from mice were cultured in complete IMDM (cIMDM) medium, which consisted of IMDM supplemented with 10% FCS, 1% penicillin/streptomycin, 20 mM HEPES (pH 7.4), 2 mM glutamine, 1 mM sodium pyruvate, 1x MEM non-essential amino acids, and 50 µM β-mercaptoethanol (all from Thermo Fisher Scientific). B16/BL6 murine melanoma cells were cultured in RPMI 1640 medium containing 10% FCS and 1% penicillin/streptomycin.

### T cell differentiation

Total CD4^+^ T cells were isolated from mouse spleens by negative selection and magnetic separation using biotinylated antibodies against CD8α, CD11b, CD19, CD45R, CD49b, and TER119 (all from BioLegend), followed by incubation with streptavidin-coated magnetic beads (BD Biosciences). Naive CD4^+^ (CD44^–^CD62L^+^CD25^–^) T cells were further purified by fluorescence-activated cell sorting (FACS) using a FACSAria II. For T cell differentiation, naive CD4^+^ T cells were stimulated for 3 days in cIMDM with plate-bound anti-CD3 (10 µg/mL, clone 145-2C11) and anti-CD28 (10 µg/mL, clone 37.51) under various T cell conditions as follows: IL-12 (10 ng/mL) and anti-IL-4 (10 μg/mL, clone 11B11) for Th1 cells; IL-4 (10 ng/mL) and anti-IFN-γ (10 μg/mL, clone XMG1.2) for Th2 cells; IL-4 (10 ng/mL), TGF-β (2 ng/mL), and anti-IFN-γ (10 μg/mL) for Th9 cells; IL-6 (40 ng/mL), TGF-β (2 ng/mL), IL-23 (20 ng/mL), anti-IFN-γ (10 μg/mL), and anti-IL-4 (10 μg/mL) for Th17 cells; IL-6 (40 ng/mL), TNF-α (20 ng/mL), IL-1β (20 ng/mL), IL-23 (20 ng/mL), anti-IFN-γ (10 μg/mL), anti-IL-4 (10 μg/mL, all BioLegend), anti-TGF-β 1,-2,-3 (5 μg/ml, clone 1D11), and FICZ (300 nM, both R&D Systems) for Th22 cells.

### Flow cytometry

Cells were stained with Zombie Aqua fixable viability dye (BioLegend) and fluorochrome-labeled anti-mouse antibodies against the following surface markers: CD3 (145-2C11), CD4 (GK1.5), CD8 (53-6.7), CD25 (PC61), CD44 (IM7), CD62L (MEL-14), CD127 (A7R34), NK1.1 (PK136), NKp46 (29A1.4), TCRβ (H57-597), and TCRγδ (GL3). Intracellular staining of T-bet (4B10), GATA3 (16E10A23), and RORγt (Q31-378) was performed using the Foxp3/Transcription Factor Staining Buffer Set (eBioscience). For intracellular cytokine staining, cells were cultured as described above and stimulated for 4 hours with phorbol 12-myristate 13-acetate (PMA, 500 ng/mL) and ionomycin (500 ng/mL, both from Sigma-Aldrich) in the presence of brefeldin A (5 µg/mL, BioLegend). Following Zombie Aqua and surface staining, cells were fixed with Cytofix/Cytoperm, permeabilized with Perm/Wash buffer (both from BD Biosciences), and then stained for the following cytokines, IFN-γ (XMG1.2), IL-2 (JES6-5H4), IL-4 (11B11), IL-9 (RM9A4), IL-17a (TC11-18H10.1), and IL-22 (Poly5164). To stimulate CD8^+^ T cells, splenocytes were incubated for 6 hours with or without IL-12 (10 ng/mL), IL-18 (100 ng/mL), PMA (500 ng/mL), and ionomycin (500 ng/mL) in the presence of brefeldin A (5 µg/mL). Cell proliferation was assessed using the CellTrace Violet Cell Proliferation Kit (Thermo Fisher Scientific). Flow cytometry was performed on a FACSCantoII (BD Biosciences), and data were analyzed using FlowJo software (TreeStar).

### RNA expression analysis

Total RNA was isolated from cells using RNAzol (Molecular Research Center) and reverse transcribed into cDNA using the PrimeScript RT Reagent Kit with gDNA Eraser (Takara Bio). Relative RNA levels were measured by real-time quantitative reverse transcription-PCR (qRT-PCR) using a StepOnePlus system (Applied Biosystems) and TB Green Premix Ex Taq II Fast qPCR (Takara Bio). The primer sequences used for qRT-PCR in this study were adopted from our previous study (31).

### RNA sequencing and data processing

For RNA sequencing (RNA-seq) analysis, total RNA was purified using the FastGene RNA Premium Kit (Nippon Genetics) and the NEBNext rRNA Depletion Kit v2 (NEB) and processed to generate cDNA using the NEBNext UltraExpress RNA Library Prep Kit (NEB, #E3330). RNA-seq libraries were prepared from the cDNA using the NEBNext Ultra II DNA Library Prep Kit (NEB, #E7645) and NEBNext Multiplex Oligos for Illumina (NEB, #E7600). Libraries with unique barcodes were pooled at equimolar ratios and sequenced with 150-bp paired-end reads on the NovaSeq platform. Sequencing reads were aligned to the mouse reference genome (mm10) using the nf-core RNA-seq pipeline (v3.18.0) with the default alignment options (STAR (v2.6.1d) and Salmon (v1.10.3)) and perform analysis of variance (ANOVA) comparisons (32). Dot plots and Volcano plots were generated using the integrated Differential Expression & Pathway analysis (iDEP v2.01).

### Chromatin immunoprecipitation

Chromatin Immunoprecipitation (ChIP) assays were performed as previously described (15). Immunoprecipitated DNA was analyzed by quantitative PCR (qPCR) using TB Green Premix Ex Taq II Fast qPCR (Takara Bio) with the following primers: ΔCBS–70F, 5´-CCCGGGAAGTAGAACGTCAC-3´ and ΔCBS–70R, 5´-AGAGGGAAATTAAACAGGTAGCCA-3´; CNS–22F, 5´-TGGGTTGCTGATGCTGTCAT-3´ and CNS–22R, 5´-CCCAGAAGAAAGAATGTTACACCC-3´; CBS + 1F, 5´-CAATGAAGCCCTATTACAGCACAG-3´ and CBS + 1R, 5´-TCTTTTAGCTGCAGGATGTACTGG-3´; CNS + 29F, 5´-CGCTCAGTATACAGCCAGTCACTT-3´ and CNS + 29R, 5´- AAGCTCTAGCTGCCCTGATTAAAA-3´; CBS + 66F, 5´-TTTGCTTACACACCAGCTGC-3´ and CBS + 66R, 5´-GGAGAGGCAAAATGATAAGATGTCC-3´.

### ChIP sequencing and data processing

ChIP sequencing (ChIP-seq) experiments were performed with 10 million cells using anti-CTCF (Cell Signaling Technology) and the SimpleChIP Enzymatic Chromatin IP Kit with magnetic beads (Cell Signaling Technology), following the manufacturer’s instructions. ChIP-seq libraries were prepared using the NEBNext Ultra II DNA Library Prep Kit (NEB) and NEBNext Multiplex Oligos for Illumina (NEB). Briefly, 70 ng of ChIP DNA fragments were end-repaired, indexed, and amplified. Libraries were sequenced with 150-bp paired-end reads on the NovaSeq platform. Reads were aligned to the mouse reference genome (mm10) using Bowtie2 (v2.5.4) and filtered with Samtools (v1.21) (33, 34). Normalized coverage tracks (CPM normalization in bigWig format) were generated for visualization using bamCoverage from deepTools (v3.5.5) (35) and the results were processed in Integrative Genomic Viewer (IGV) (v2.16). Peak calling was performed using MACS3 (v3.0.2) and R using the rtracklayer package (36). Motif enrichment analysis was performed using findMotifsGenome.pl in the HOMER suite (v4.10.4) (37).

### Region capture micro-c and data processing

Region Capture Micro-C (RCMC) was performed as previously described (38, 39). Briefly, 5 million cells were doubly crosslinked with 3 mM disuccinimidyl glutarate (Thermo Fisher Scientific) and 1% formaldehyde (Nacalai Tesque). Intact nuclei were extracted with Micro-C Buffer #1 (MB#1; 50mM NaCl, 10mM Tris-HCl pH 7.5, 5mM MgCl_2_, 1mM CaCl_2_, 0.2% NP-40, 1x Protease Inhibitor Cocktail) and digested with 20 U of MNase (Worthington BioChem) in 500 µL of MB#1 at 37 °C for 10 minutes. Digested chromatin was washed twice with MB#2 (50mM NaCl, 10mM Tris-HCl pH 7.5, 10mM MgCl_2_) and processed using the NEBNext End Repair Module (NEB) at 37 °C for 15 minutes. To generate 5´ fragment overhangs for end-blunting and labeling, 50 U of Klenow Fragment (NEB) was added and the sample were incubated at 37 °C for 15 minutes. Next, a mixture of dNTPs in end-labeling buffer, containing 66 µM each of dTTP, dGTP, biotin-dATP (Jena Bioscience), and biotin-dCTP (Jena Bioscience) was added and the sample were incubated at room temperature for 45 minutes. The samples were washed with MB#3 (50 mM Tris-HCl pH7.5, 10 mM MgCl_2_, and 100 µg/mL BSA), and proximity ligation was performed in a ligation reaction (10,000 U of T4 DNA Ligase, 1x T4 DNA Ligase buffer, 100 µg/mL of BSA) at 25 °C overnight. To remove biotinylated dNTPs from all unligated fragment ends, the samples were digested by 1,000 U of Exonuclease III (NEB) at 37 °C for 15 minutes. To prepare ligated DNA for library preparation, dinucleosome-sized DNA fragments (250–400 bp) were gel-extracted and purified with Dynabeads MyOne Streptavidin C1 (Thermo Fisher Scientific). Illumina library preparation was performed using the NEBNext Ultra II kit (NEB) and NEB Multiplex Oligos for Illumina. The capture locus (mm10, chr10:118,369,795–118,508,916) was tiled end-to-end with a set of 120-mer probes, and capture enrichment was performed according to the xGen Hybrid Capture protocol (Integrated DNA Technologies). Pooled libraries were sequenced with 150 bp paired-end reads on the NovaSeq (Illumina). Valid RCMC contact read pairs were obtained from the HiC-Pro (v3.1.0) analysis pipeline (40). Contact matrices were normalized with ICE (iterative correction and eigenvector decomposition) method (41) to correct for coverage bias and quantified with the HiCCUPS algorithm implemented in the JUICER package (v1.11.04), as previously described (42). For visualization with Juicebox (v2.15), valid pairs were converted into.hic files using the “hicpor2juicebox.sh” script (43). Contact matrices were generated at 1 kb bin resolution, enabling robust detection of TAD-scale interactions. All RCMC experiments were performed with two independent biological replicates, and consistent results were obtained across replicates.

### Chromosome conformation capture

Quantitative chromosome conformation capture (3C) assays were performed as previously described (15), using *Bam*HI and *Bgl*II for enzymatic digestion of chromatin derived from Th1 cells. The 3C samples were analyzed using PrimeTime real-time qPCR (Integrated DNA Technologies). As a control template, equimolar amounts of four BAC clones spanning the mouse *Ifng* locus (B6Ng01-263N13, B6Ng01-295L21, B6Ng01-161P12, and B6Ng01-333J24; RIKEN Bioresource Research Center) and a BAC spanning the mouse *Actb* locus (B6Ng01-258D04) were mixed and then digested and ligated. Ligation frequencies between the analyzed pairs were normalized to those detected between two restriction fragments in the *Act*B locus. Each 3C assay was performed with three independent biological replicates, with technical triplicates per sample. Statistical significance of differences in ligation frequencies was assessed using a non-paired two-tailed Student’s *t*-test. The primers and PrimeTime probes used for real-time PCR were as follows: *Ifng*–70 (anchor), 5´-TGGTGCTGGCGAAAGTCTTT-3´; *Ifng*–34, 5´-CGCGGAATTACCCCCAAGTA-3´; *Ifng*–22, 5´-GCTTTGATCATAGCTTCATTATCCT-3´; *Ifng*-promoter, 5´-CAGATGTAAGATGGGATCTCAGAGA-3´; *Ifng* + 18/20, 5´-ACTCCAACGGGGCAACTAAG-3´; *Ifng* + 46, 5´-AGGGAAAATGCCAGCTCTGA-3´; *Ifng* + 66, 5´-CACACTGTAGTTAGGGAGGTCA-3´; *Ifng* + 104, 5´-ACTTTGGCCTCTCAAGGGAA-3´; *Ifng*ΔCBS–70-probe, 5´-FAM-AGGCTGGCCTCGAACTCCGAAAC-IBFQ-3´; *Actb*-1, 5´-TGGCATTGTTACCAACTGGGA-3´; *Actb*-2, 5´-GTCTAAGTGGAGCCCCTGTC-3´; *ActB*-probe, 5´-FAM-AGACATGCAAGGAGTGCAAGAACACAGC-IBFQ-3´.

### *Cryptococcus neoformans* infection

*Cryptococcus neoformans* (*C. neoformans*) strain B3501 was grown on potato dextrose agar (PDA) plates for 2 days, washed, and suspended in sterile PBS. Mice were infected intraperitoneally (i.p.) with 500 µL of a suspension containing 1.5x10^7^ cells/mL, as previously described (44). To measure fungal burden, mice were euthanized, and spleen and lung homogenates were plated on PDA plates. After a 2-day incubation period, colony-forming units (CFU) were counted. Fungal burden foreach organ was expressed as CFU/mL/mg of tissue. To measure IFN-γ production from spleen cells after infection, single-cell suspensions were prepared from spleens and cultured in 24-well tissue culture plates at 2 x 10^6^ cells/well in the presence of concanavalin A (Con A; 5 µg/mL). Mouse serum was collected, and IFN-γ was measured using an ELISA MAX Deluxe Set (BioLegend). These measurements were conducted 7 days after infection.

### Melanoma lung metastasis

An intravenous mouse melanoma model was established as previously describes (45). Briefly, B16BL6 cells were harvested, washed, and resuspended in PBS Then, 2x10^5^ cells were intravenously injected into the tail vein of syngeneic C57BL/6 mice. After 21 days, lungs were excised, and visible metastatic colonies on the lungs were photographed and counted.

### T cell-induced colitis

Naive (CD25^–^CD45RB^+^) CD4^+^ T cells were purified by flow cytometric sorting from wild-type (WT), ΔCBS–70, or ΔCBS+66 mice. Age- and sex-matched syngeneic *Rag2* ^–/–^ mice were injected i.p. with 7x10^5^ naive T cells. Mice were observed daily, and weight loss was monitored over 6–8 weeks. The entire colon was resected from each mouse, and its length from the cecum to the anus was measured before dissection for histological assessment and flow cytometry. For histological assessment of colitis severity, colon pieces were fixed in buffered 10% formalin and embedded in paraffine. Five-µm thin sections were cut and stained with hematoxylin and eosin. To measure IFN-γ production from lamina propria cells, single-cell suspensions were prepared from colon pieces.

### Histology

Mouse tissues were fixed in 10% formalin for 48 hours, preserved in 70% ethanol, and embedded in paraffin. The paraffin blocks were cut into 5-µm longitudinal sections and stained with hematoxylin and eosin (H&E).

### Next-generation sequencing data and processing

The raw data generated in this study have been deposited in the Gene Expression Omnibus (GEO) under the following accession numbers: GSE300739 (CTCF ChIP-seq), GSE300740 (RNA-seq), and GSE300741 (Hi-C). In addition, previously published NGS datasets were utilized in this study. Specifically, Hi-C, CTCF ChIP-seq, and H3K27ac ChIP-seq datasets were obtained from the GEO database (accession number GSE215181) as previously reported by Liu et al. (13). These datasets had already been processed and analyzed in the original publication, and no additional bioinformatics reanalysis was performed in the present study. For RNA-seq data processing, reads were aligned to the mm10 reference genome using the nf-core RNA-seq pipeline (v3.18.0) (46), with STAR (v2.6.1d) (32) and Salmon (v1.10.3) (47). Differential expression analysis and visualization (dot plots, volcano plots) was performed using iDEP (v2.01) with ANOVA framework (48). For ChIP-seq data processing, reads were aligned to mm10 with Bowtie2 (v2.5.4) (33), filtered with Samtools (v1.21) (34), and normalized bigWig tracks were generated using deepTools bamCoverage (v3.5.5) (35). Visualization was performed with Integrative Genomics Viewer (IGV) (v2.16) (49). Peak calling was performed with MACS3 (v3.0.2) (50), and motif enrichment was analyzed with HOMER (findMotifsGenome.pl) (v4.10.4) (37). For Hi-C/RCMC data processing, valid contact pairs were obtained with HiC-Pro (v3.1.0) (40), normalized using the ICE method (41), and converted into.hic files for visualization with Juicebox (v2.15) (43). Although automated loop-calling algorithms such as cLoops2 (v0.1.0) (51) and HiCCUPS (42) were tested, robust detection of focal interactions was not achieved. Instead, interaction strength was quantified by extracting bin-level counts directly from Juicebox (v2.15) contact maps at 1 kb resolution, following an approach similar to Petermann et al. (52). All RCMC experiments were performed with at least two independent biological replicates, and consistent results were obtained across replicates.

### Statistical analysis

Significance was assessed using non-paired two-tailed Student’s t test.

## Results

### Topological organization and regulatory landscape of the *Ifng* locus

**Figure 1:**
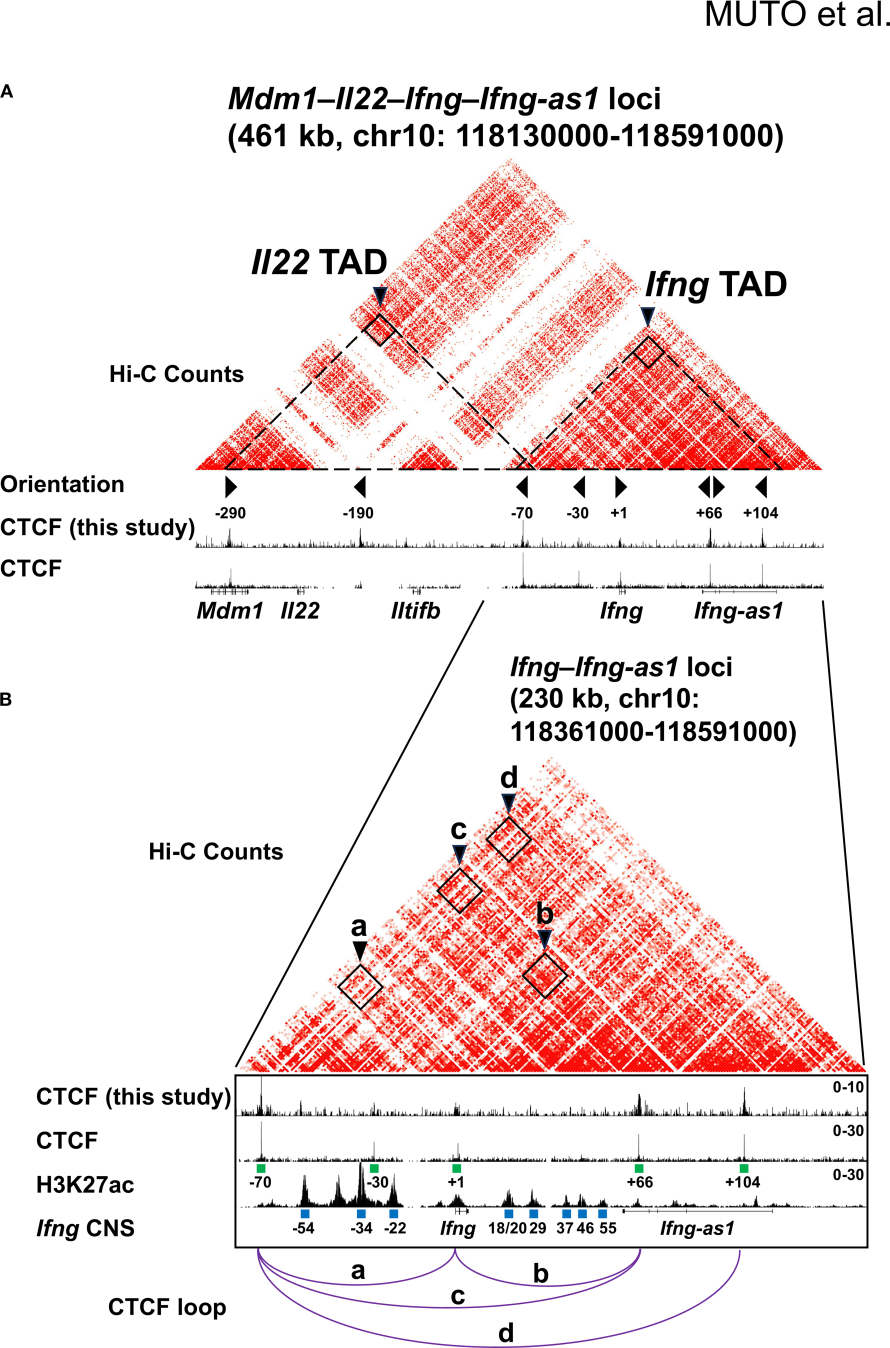
CTCF-mediated chromatin architecture at the *Mdm1–Il22–Iltifb–Ifng–Ifng-as1* loci. **(A, B)** Heatmaps depicting Hi-C chromatin interactions and epigenomic features at the *Ifng* locus in Th1 cells. CTCF ChIP-seq peaks generated in this study are shown together with published CTCF ChIP-seq and H3K27ac ChIP-seq datasets reported by Liu et al. (13). Hi-C data were also obtained Liu et al. (13). Putative *cis*-regulatory elements (blue rectangles) were defined based on H3K27ac density and annotated by their position relative to the *Ifng* transcription start site (TSS) (− upstream, + downstream). CTCF motifs are marked with green rectangles, and their orientation is shown by triangle markers. CTCF-bound TAD boundaries are indicated by arrowheads in **(A)** and chromatin loops by arrowheads and purple lines in **(B)**.

Our previous work revealed that the mouse *Ifng* locus is insulated from neighboring genes (*Mdm1*, *Il22*, *Iltifb*, and *Dyrk2*) by CTCF-binding boundary elements located –70 kb upstream and +66 kb downstream of the *Ifng* transcription start site (TSS) in Th1 cells (15). The *Ifng* –70 kb and +66 kb CBSs interact with each other as well as with an intragenic CBS located +1 kb from the TSS (**Figure 1B**, arrowheads a, b, and c), suggesting that these CBSs act as chromatin loop anchors. More recent *in situ* Hi-C contact maps have shown that the *Ifng* locus resides within a much larger chromatin interaction domain than previously appreciated (13, 52). Specifically, *Ifng* and the adjacent long noncoding RNA, *Ifng-as1* are located within a 192-kb TAD, hereafter referred to as the *Ifng* TAD, which displays robust self-interactions between the –70 kb and +104 kb CBSs (**Figures 1A, B**, arrowhead d). A neighboring 230-kb TAD, referred to as the *Il22* TAD, further separates *Mdm1*, *Il22*, and *Iltifb* from the *Ifng* TAD (**Figure 1A**). These domains are demarcated by a prominent boundary containing the convergent *Ifng* –70 kb CBS, while the *Ifng* + 66 kb CBS likely functions as an intra-TAD boundary within the *Ifng* TAD.

**Figure 2:**
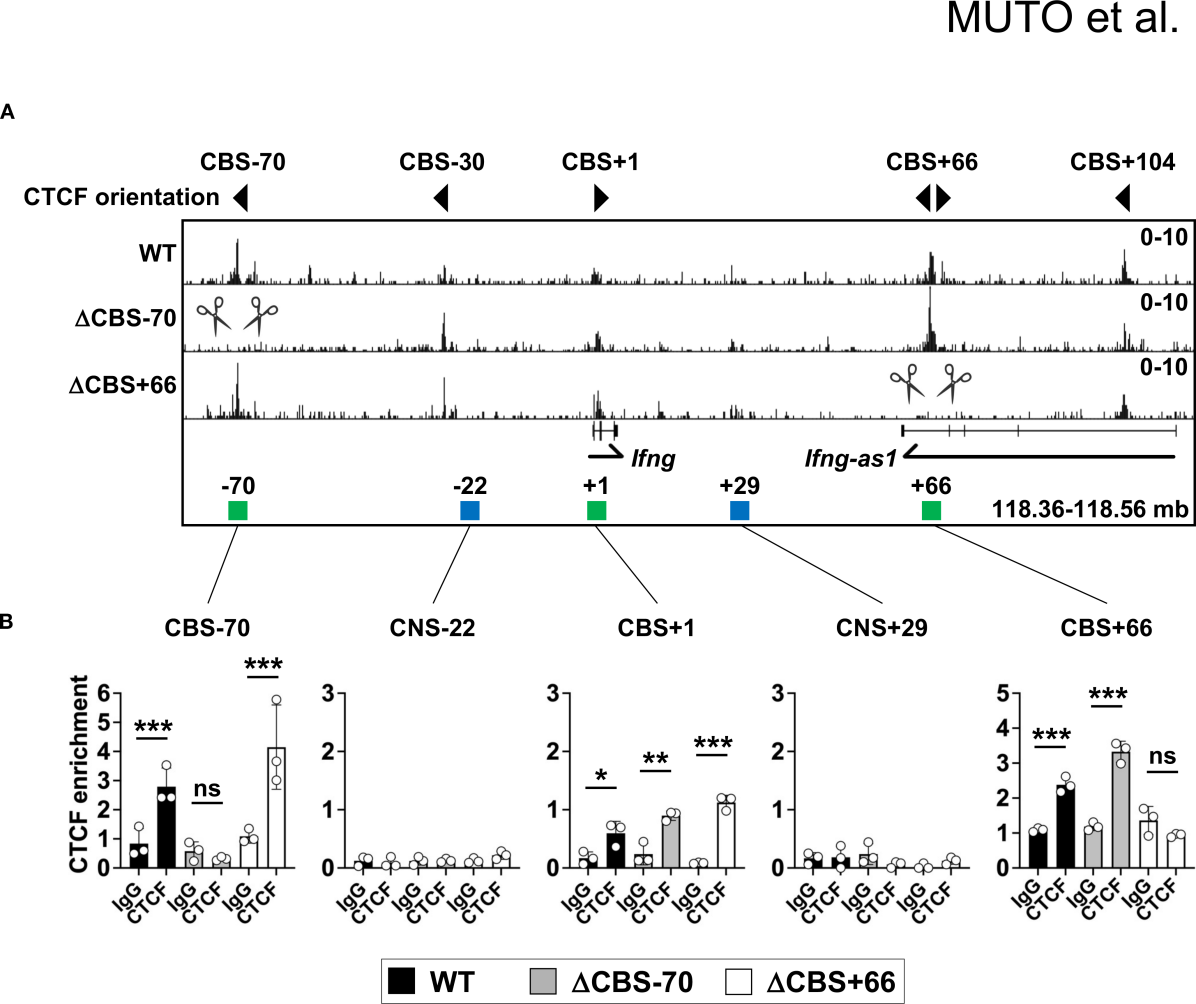
Disruption of CTCF binding motifs abolishes CTCF binding at the *Ifng* locus. **(A)** Genome browser tracks of CTCF ChIP-seq signals at the *Ifng* locus in WT, ΔCBS–70, and ΔCBS+66 Th1 cells. Local genes, CBSs (green rectangles), and enhancers (blue rectangles) are annotated. Triangle markers indicate motif orientations. CBSs targeted for deletion by CRISPR-Cas9 are marked by scissors. Data represent at least two independent experiments. **(B)** ChIP-qPCR analysis of CTCF or control IgG binding at the *Ifng* locus in WT, ΔCBS–70, and ΔCBS+66 Th1 cells, normalized to input. Bar graphs show mean ± SD. Significance: non-paired two-tailed Student’s t test. *p < 0.05; **p < 0.01; ***p < 0.001; ns, not significant.

To investigate the functional role of CTCF occupancy at the *Ifng* locus in IFN-γ production and immune function, we generated *Ifng* ΔCBS–70 and ΔCBS+66 mice using CRISPR-Cas9 genome editing (**Supplementary Figures 1A, B**). Both strains were viable, showed normal Mendelian inheritance, and displayed no overt developmental abnormalities. ChIP-seq and ChIP-qPCR confirmed the loss of CTCF binding at the targeted CBSs in Th1 cells from each respective mutant line, while CTCF occupancy at other CBSs within the *Ifng* TAD, including the –30 kb, +1 kb, and +104 kb sites, remained unaffected (**Figures 2A, B**).

### Loss of the *Ifng* –70 kb CBS, but not the *Ifng* + 66 kb CBS, impairs IFN-γ production in Th1 cells

**Figure 3:**
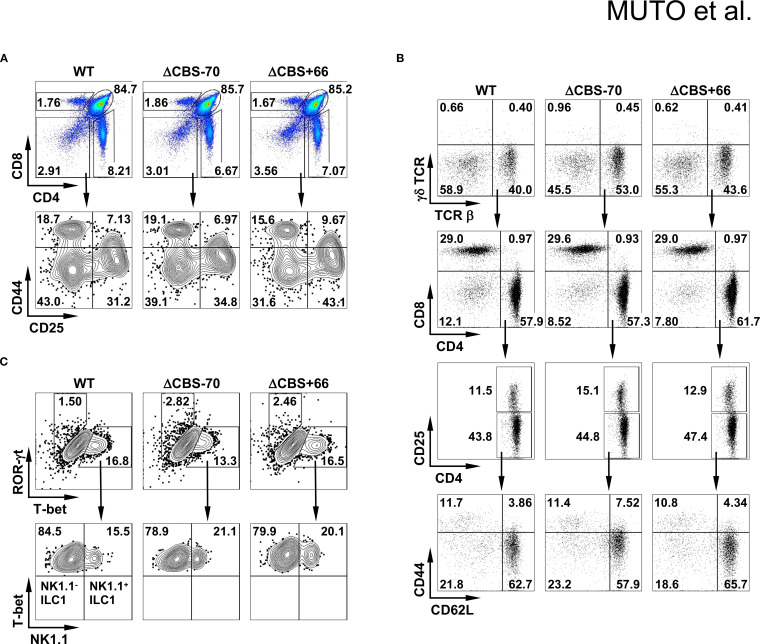
Deletion of *Ifng* CBSs does not alter lymphocyte subsets or T cell development. **(A, B)** Flow cytometric analysis of cells from thymus **(A)** and lymph node **(B)** of WT, ΔCBS–70, and ΔCBS+66 mice. **(C)** Flow cytometric analysis of innate lymphoid cells (ILCs) isolated from the small intestine. Data represent three independent experiments.

**Figure 4:**
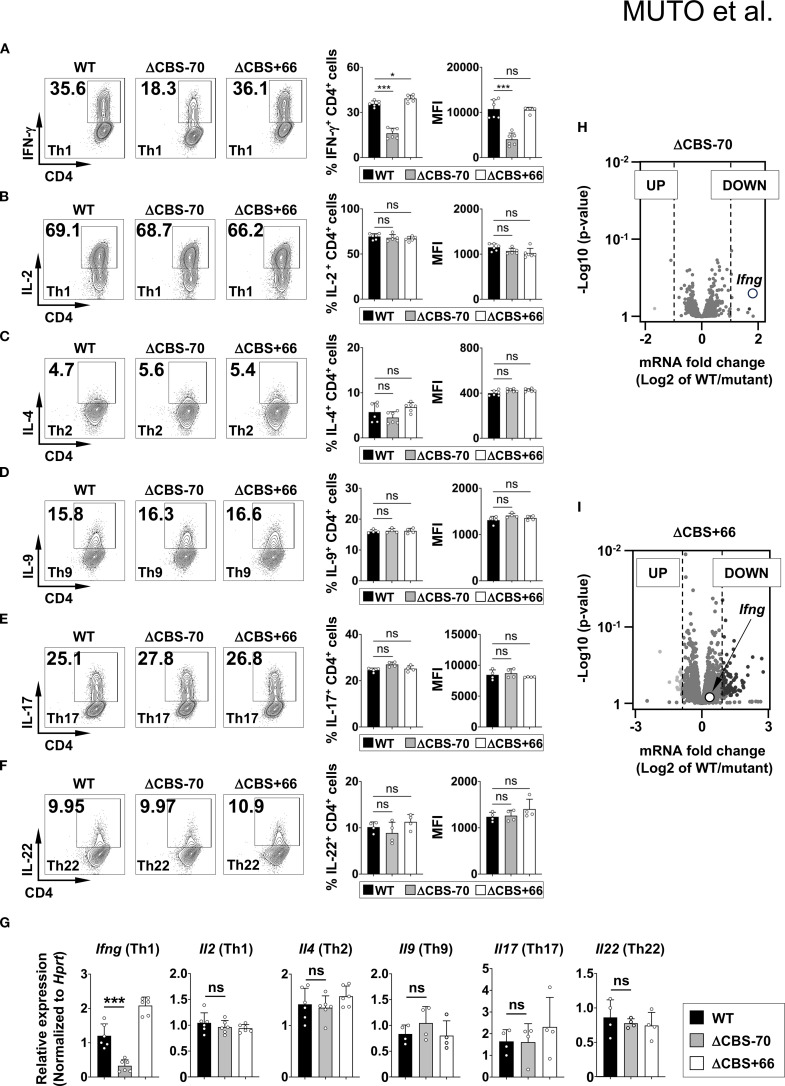
Loss of the –70 kb CBS reduces *Ifng* expression in Th1 cells. **(A–F)** Naive CD4^+^ T cells from WT, ΔCBS–70, and ΔCBS+66 mice were FACS-sorted and cultured under Th1 **(A, B)**, Th2 **(C)**, Th9 **(D)**, Th17 **(E)**, and Th22 **(F)** polarization conditions for 3 days. Cytokine production was assessed by intracellular staining and flow cytometry. **(G)** *Ifng* mRNA levels were analyzed by real-time qPCR on day 3 and normalized to *Hprt*. **(H, I)** Volcano plots showing differentially expressed genes from-RNA-seq data in Th1 cells comparing WT vs. ΔCBS–70 **(H)** and WT vs. ΔCBS+66 **(I)**. Genes with a log_2_ fold change >1 were considered significantly altered. WT, n = 3; ΔCBS–70, n = 3; ΔCBS+66; n = 3. Bar graphs show mean ± SD. Significance: non-paired two-tailed Student’s t test. *p < 0.05; **p < 0.01; ***p < 0.001; ns, not significant.

Both ΔCBS–70 and ΔCBS+66 mice exhibited normal T cell development in primary and secondary lymphoid organs. Flow cytometric analyses of T cell populations in the thymus and lymph nodes, as well as innate lymphoid cells (ILCs) in the small intestinal lamina propria, revealed no significant differences compared to WT mice (**Figures 3A–C**). To assess the role of CTCF binding at the *Ifng* –70 kb and +66 kb CBSs in IFN-γ production, we sorted naive CD4^+^ T cells from WT and mutant mice and differentiated them *in vitro* into Th1, Th2, Th9, Th17, and Th22 effector lineages for 3 days to mimic early Th cell polarization. Following stimulation with phorbol 12-myristate 13-acetate (PMA) and ionomycin, ΔCBS–70 Th1 cells showed a significant reduction in IFN-γ expression at both the protein and mRNA levels compared to WT Th1 cells (**Figures 4A, G**). In contrast, *Ifng* expression in ΔCBS+66 Th1 cells was comparable to that in WT cells (**Figures 4A, G**). Importantly, cell proliferation (**Supplementary Figure 2A**) and the production of other lineage-associated cytokines, including those from Th2, Th9, Th17, and Th22 cells, were unaffected in both mutant lines (**Figures 4C–G**). The expression of IL-2 and the Th1 master regulator T-bet also remained unchanged in both WT and mutant Th1 cells (**Figures 4B, G**, **Supplementary Figure 2B**).

To corroborate these findings, we performed RNA-seq of WT and mutant Th1 cells cultured for 3 days *in vitro*. Consistent with the flow cytometry results, *Ifng* was the most significantly downregulated gene exclusively in ΔCBS–70 Th1 cells (**Figures 4H, I**). Only a limited number of additional genes were differentially expressed, indicating that deletion of the *Ifng* –70 kb CBS exerts a localized, rather than global, transcriptional effect.

**Figure 5:**
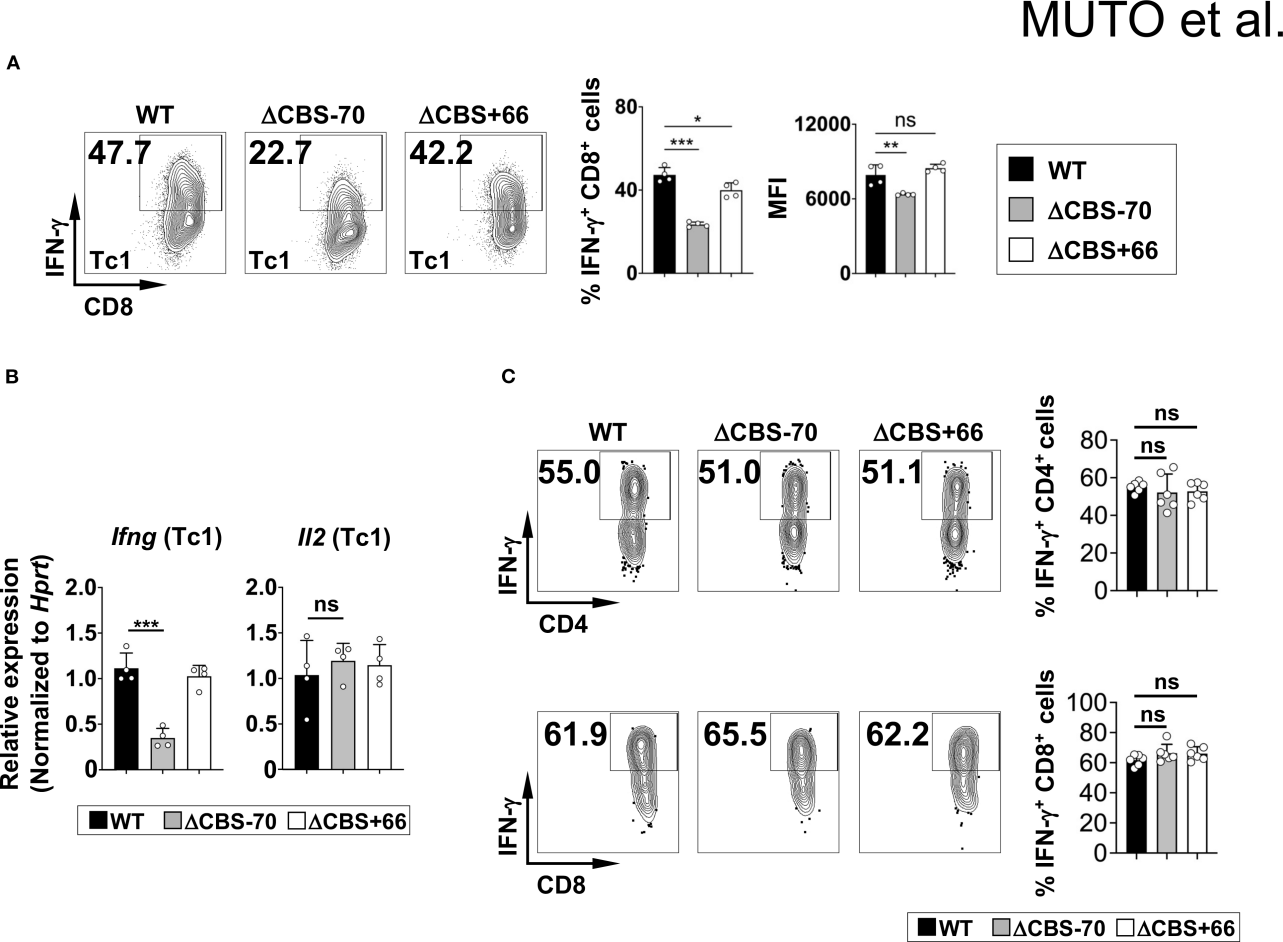
The –70 kb CBS is required for *Ifng* expression in CD8^+^ T cells but not in memory T cells. **(A, B)** CD3^+^ T cells from WT, ΔCBS–70, and ΔCBS+66 mice were cultured with IL-12 for 3 days. **(A)** IFN-γ production in CD8^+^ T cells assessed by flow cytometry. **(B)** *Ifng* mRNA expression in CD8^+^ T analyzed by real-time qPCR and normalized to *Hprt*. **(C)** Representative flow plots showing IFN-γ production in effector memory CD4^+^ and CD8^+^ T (CD25^–^CD44^+^CD62L^–^) cells. Bar graphs show mean ± SD. Significance: non-paired two-tailed Student’s t test. *p < 0.05; **p < 0.01; ***p < 0.001; ns, not significant.

Given this critical role of the *Ifng* –70 kb CBS in early Th1 cells, we next evaluated IFN-γ production in other IFN-γ-producing T cell subsets. Upon PMA/ionomycin stimulation, CD8^+^ T cells from ΔCBS–70 mice also produced significantly less IFN-γ compared to WT mice, whereas ΔCBS+66 CD8^+^ T cells exhibited normal IFN-γ production (**Figures 5A, B**). In contrast, IFN-γ levels in effector memory CD4^+^ and CD8^+^ T cells were similar among WT and mutant mice (**Figure 5C**), consistent with prior findings (13). These results suggest that the *Ifng* –70 kb CBS is dispensable in memory T cells, but critically essential during the initiation and stabilization phases of Th1 differentiation, when high levels of IFN-γ are required.

### CTCF occupancy at the *Ifng* –70 kb CBS, but not at the *Ifng* + 66 kb CBS, promotes the *Ifng* promoter–enhancer interactions

**Figure 6:**
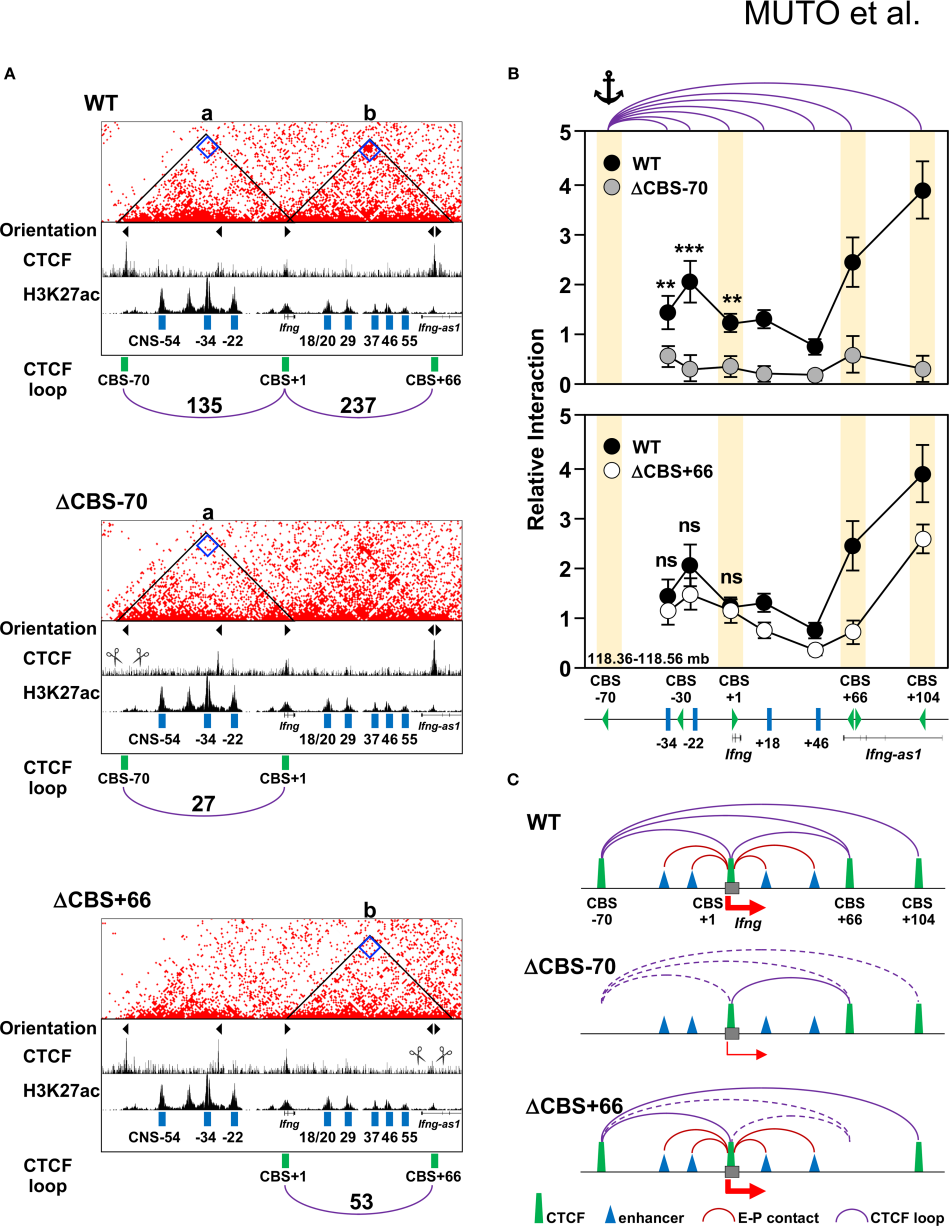
The –70 kb CBS mediates enhancer–promoter interactions at the *Ifng* locus. **(A)** RCMC interaction heatmaps showing chromatin interactions in Th1 cells from WT, ΔCBS–70, and ΔCBS+66 mice. Valid pairs were processed with HiC-Pro (v3.1.0), normalized using the ICE method to correct for coverage bias, and converted into.hic files for visualization in Juicebox (v2.15). Contact matrices are displayed at 1-kb resolution. The resolution and color scale were optimized to highlight interactions at the –70 kb and +66 kb CBSs. Triangle markers indicate motif orientations. CTCF and H3K27ac ChIP-seq profiles (generated in this study and from public datasets) are shown below. Automated loop-calling algorithms such as cLoops2 and HiCCUPS were tested but did not yield robust calls for focal interactions at this locus. Therefore, loop strength was instead quantified by extracting bin-level counts at 1-kb resolution directly from Juicebox contact maps, following the same procudure described in Materials and Methods. Loop read counts are indicated on arches. **(B)** 3C-qPCR analysis of chromatin looping used the –70 kb BamHI/BglII fragments as the anchor. Interaction frequencies were quantified at single restriction fragment resolution and normalized to the *Actb* locus. Comparisons are shown for WT vs. ΔCBS–70 (top) and WT vs. ΔCBS+66 (bottom). Each 3C analysis was performed in three independent biological replicates, with technical triplicates per sample. CBSs are shown as yellow rectangles; anchor sites are marked. **(C)** Schematic diagrams summarizing chromatin architecture in WT and mutant Th1 cells. Solid arched lines represent maintained loops; dashed arched lines represent disrupted interactions. Significance: non-paired two-tailed Student’s t test. **p < 0.01; ***p < 0.001; ns, not significant.

We have previously identified several enhancers within the *Ifng* locus, namely CNS–34, CNS–22, CNS + 18/20, and CNS + 29, which are marked by H3K27ac (**Figure 1B**) and contribute to *Ifng* transcription during Th1 differentiation (12). According to the enhancer–promoter looping model, long-range interactions between enhancers and their target promoters are often stabilized by CTCF-mediated chromatin loops anchored at CBSs located at TAD boundaries or within TADs (22). To investigate whether the CBSs at the *Ifng* locus contribute to chromatin architecture that supports gene regulation, we performed Region-Capture Micro-C (RCMC) to generate chromatin contact maps for WT, ΔCBS–70, and ΔCBS+66 Th1 cells. Visual inspection of these maps revealed that deletion of the *Ifng* –70 kb CBS markedly reduced chromatin interactions between the deleted CBS region and the *Ifng* gene body in ΔCBS–70 Th1 cells (**Figure 6A**, loop a), correlating with decreased *Ifng* expression (**Figures 4A, G, H**). Similarly, the *Ifng* + 66 kb CBS showed reduced contact with the *Ifng* locus in ΔCBS+66 Th1 cells (**Figure 6A**, loop b); however, deletion of this CBS did not impair *Ifng* expression (**Figures 4A, G, I**), suggesting that the +66 kb site is not required for transcriptional activation.

To further delineate these chromatin interactions, we performed 3C analysis using the *Ifng* –70 kb CBS as the anchor site, and confirmed a prominent interaction between the –70 kb and +104 kb CBSs in WT Th1 cells (**Figure 6B**), consistent with recent *in situ* Hi-C data showing that these sites demarcate the 192-kb *Ifng* TAD (13, 52). Importantly, deletion of the *Ifng* –70 kb CBS substantially weakened long-range chromatin interactions between this region and a broad segment surrounding *Ifng* (–34 kb to +104 kb), encompassing the promoter and multiple enhancers, in ΔCBS–70 Th1 cells (**Figure 6B**, top panel). In contrast, ΔCBS+66 Th1 cells showed no significant changes in chromatin looping relative to WT Th1 cells (**Figure 6B**, bottom panel), indicating that the *Ifng* + 66 kb CBS does not function in the *Ifng* enhancer–promoter interactions or disrupt overall *Ifng* TAD integrity. Together, these findings support a model in which CTCF binding at the –70 kb CBS facilitates stable chromatin looping with the +104 kb CBSs, thereby promoting enhancer–promoter contacts required for robust *Ifng* expression during Th1 cell differentiation (**Figure 6C**).

### CTCF occupancy at the *Ifng* –70 kb CBS, but not at the *Ifng* + 66 kb CBS, is necessary for Th1 responses during host defense and tumor immunosurveillance

**Figure 7:**
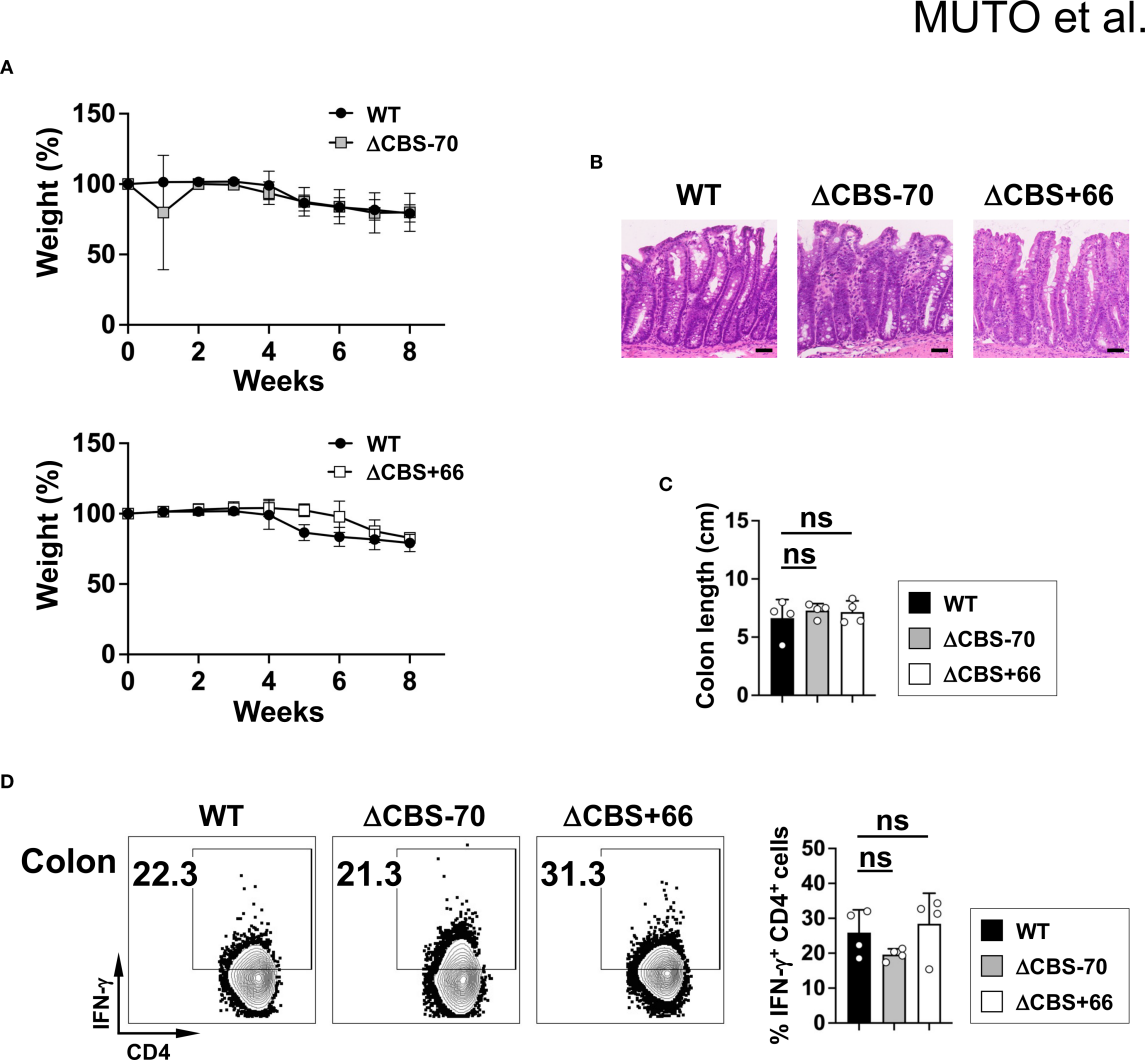
CBS deletions do not affect colitis severity. **(A)** Body weight changes in *Rag2* ^–/–^ mice receiving naive CD4^+^CD25^–^CD45RB^+^ T cells from WT, ΔCBS–70, or ΔCBS+66 mice. **(B)** Hematoxylin and eosin (H&E) staining of colon sections at 8 weeks post-transfer. Scale bars, 50 µm. **(C)** Colon lengths measurements. **(D)** IFN-γ production by lamina propria CD4^+^ T cells assessed by flow cytometry. Data represent three independent experiments. Bar graphs show mean ± SD. Significance: non-paired two-tailed Student’s t test. ns, not significant.

To further investigate the *in vivo* role of CTCF-mediated chromatin architecture at the *Ifng* locus, we first employed a T cell transfer colitis model of chronic autoimmune inflammation, in which both IFN-γ and IL-17 contribute synergistically to disease pathogenesis. Transfer of CD4^+^CD25^–^CD45RB^+^ T cells from either WT or mutant mice induced comparable disease severity, as evidenced by weight loss, colon shortening, and histological scores (**Figures 7A–C**). Moreover, T cells isolated from the colonic lamina propria of WT and mutant mice exhibited similar levels of IFN-γ production (**Figure 7D**), indicating that CTCF-mediated chromatin architecture at the *Ifng* locus does not significantly affect IFN-γ expression in this chronic inflammatory context.

**Figure 8:**
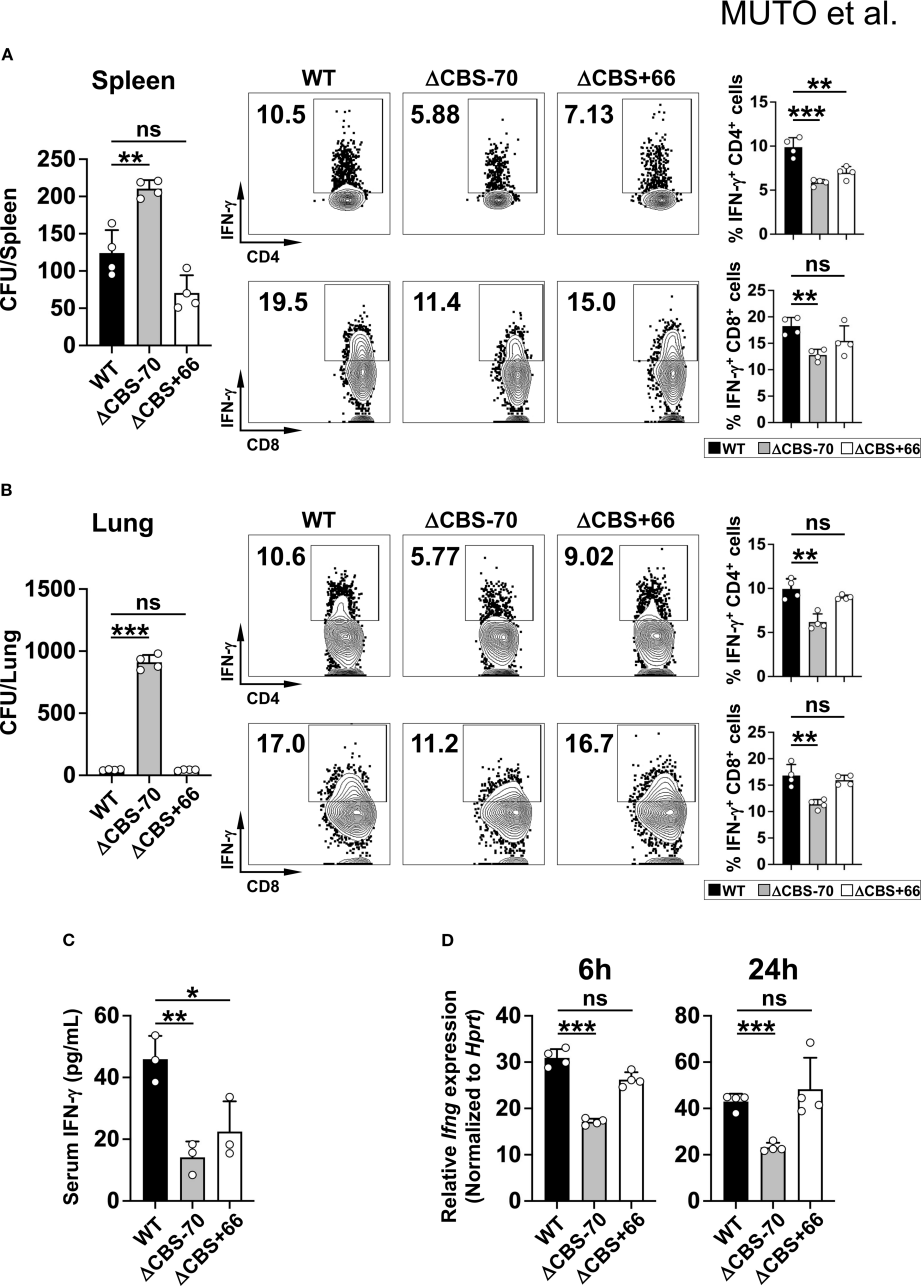
ΔCBS–70 mice exhibit impaired resistance to cryptococcal infection. WT, ΔCBS–70, and ΔCBS+66 mice were infected intraperitoneally with 8x10^6^ **(*C*)** *neoformans* (B3501 strain) cells. **(A, B)** CFU counts in the spleens **(A)** and lungs **(B)** on day 7 post-infection. IFN-γ production in CD4^+^ and CD8^+^ T cells from these organs was analyzed by flow cytometry. **(C)** Serum IFN-γ levels measured by ELISA. **(D)** Splenocytes cultured with Con A (1 µg/ml) for 6 h or 14 h; *Ifng* expression was analyzed by real-time qPCR and normalized to *Hprt*. Bar graphs show mean ± SD. Significance: non-paired two-tailed Student’s t test. *p < 0.05; **p < 0.01; ***p < 0.001; ns, not significant.

Given the lack of phenotype in the colitis model, we next examined the role of CTCF-bound CBSs in an acute infection setting a *C. neoformans* model, where both innate and adaptive immune cell-derived IFN-γ are essential for host protection (44). Although no mortality was observed during the 7-day infection period, ΔCBS–70 mice exhibited significantly reduced serum IFN-γ levels compared to WT and ΔCBS+66 mice (**Figure 8C**). This reduction was accompanied by increased fungal colony-forming units (CFUs) in the spleen and lung of ΔCBS–70 mice (**Figures 8A, B**). Flow cytometric analysis further revealed decreased IFN-γ production by CD4^+^ and CD8^+^ T cells in the spleen and lung of ΔCBS–70 mice, whereas IFN-γ production in ΔCBS+66 mice was comparable to that in WT mice (**Figures 8A, B**). Consistent with this, spleen cells isolated from infected ΔCBS–70 mice produced significantly less *Ifng* mRNA than those from WT or ΔCBS+66 mice after stimulation with ConA at both 6 and 24 hours (**Figure 8D**).

**Figure 9:**
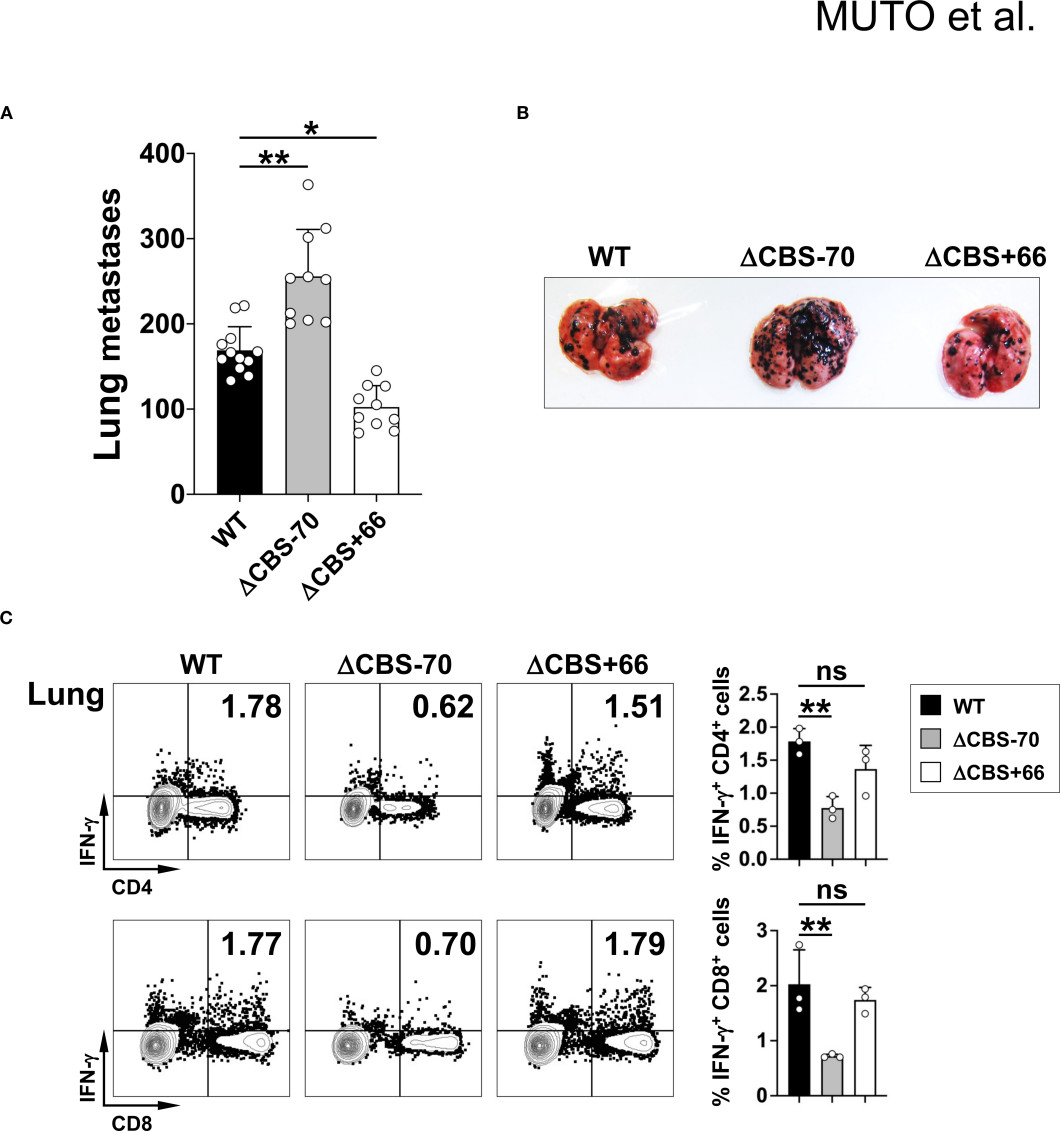
Loss of the –70 kb CBS increases pulmonary metastasis. B16BL6 cells (5x10^5^) were intravenously injected into WT, ΔCBS–70, and ΔCBS+66 mice. **(A)** Quantification of lung metastatic foci 21 days post-injection. **(B)** Representative lung images showing black metastatic foci. **(C)** IFN-γ production in lung CD4^+^ and CD8^+^ T cells analyzed by flow cytometry. Bar graphs show mean ± SD. Significance: non-paired two-tailed Student’s t test. *p < 0.05; **p < 0.01; ***p < 0.001; ns, not significant.

Since CD4^+^ and CD8^+^ T cells are the primary sources of IFN-γ in the adaptive immune system and play key roles in anti-tumor immunity, we next evaluated the functional impact of CBS deletions in a lung metastasis model of tumor surveillance. Following intravenous injection of B16 melanoma cells and a 21-day incubation period, ΔCBS–70 mice developed significantly more pulmonary tumor nodules than either WT or ΔCBS+66 mice (**Figures 9A, B**). Correspondingly, CD4^+^ and CD8^+^ T cells recovered from tumor-bearing lungs of ΔCBS–70 mice produced significantly less IFN-γ than those from WT and ΔCBS+66 mice (**Figure 9C**). Taken together, these *in vivo* findings, consistent with our *in vitro* data, demonstrate that deletion of the *Ifng* –70 kb CBS, but not the *Ifng* + 66 kb CBS, severely impairs IFN-γ expression and Th1-mediated immune responses needed for host defense against infection and effective anti-tumor immunity.

## Discussion

### The hierarchical role of chromatin architecture in *Ifng* regulation

Recent studies underscore the intimate link between chromatin architecture and transcriptional regulation, particularly at the level of TADs and intra-TADs (21–25). The *Ifng* locus, encompassing *Ifng*, *Ifng-as1*, and several enhancers (CNS–34, CNS–22, CNS + 18/20, and CNS + 29), is flanked by two TAD boundaries marked by the –70 kb and +104 kb CBSs, which together define the broader *Ifng* TAD (13, 52). These CNS elements have been functionally validated as enhancers by reporter assays (9–12), and their roles are further supported by enrichment of the active histone modification H3K27ac at these loci (13). Thus, the chromatin loops we observed in this study connect previously characterized functional enhancers to the *Ifng* promoter, highlighting the physiological relevance of these regulatory interactions.

Within this domain, the *Ifng* gene and its associated enhancers are further organized into an intra-TAD structure delimited by the –70 kb and +66kb CBSs. Although the 3D topology of the *Ifng* locus has been characterized, the specific contributions of these chromatin domains to enhancer-mediated *Ifng* expression have remained unclear. In this study, we demonstrated that CTCF occupancy at the *Ifng* locus regulates gene expression by modulating the accessibility of enhancers to the promoter. Our RCMC and 3C analyses revealed that CTCF binding at the –70 kb CBS is essential for forming a loop between the –70 kb and +104 kb CBSs, thereby organizing the *Ifng* TAD and facilitating enhancer–promoter interactions. Notably, deletion of the +66 kb CBS located at an intra-TAD boundary did not impact *Ifng* expression, despite disruption of intra-TAD interactions. This suggests that the overarching TAD structure alone is sufficient to enable proper enhancer–promoter communication at the *Ifng* locus, and that the intra-TAD boundary at +66 kb is not essential for transcription. This observation contrasts with findings at other loci, such as *HoxD* and *Thy1*, where intra-TAD-dependent interactions are indispensable for gene expression (53, 54). Thus, our findings support a model in which chromatin architecture is hierarchically organized: TADs provide a permissive framework for gene regulation, while intra-TAD structures may refine enhancer–promoter interactions in a context-dependent manner.

Importantly, we also considered the potential impact of CBS deletions on neighboring non-coding RNAs. In our RNA-seq analysis of Th1 cells at day 3 of differentiation, expression of *Ifng-as1* was extremely low and below the detection threshold, and was not significantly altered in ΔCBS–70 or ΔCBS+66 cells. Therefore, the transcriptional changes reported here are unlikely to be confounded by *Ifng-as1* at this early stage. This is consistent with observations by Liu et al., who similarly reported minimal *Ifng-as1* expression at early differentiation time points, but noted a reduction in *Ifng-as1* expression in CBS-deficient mice at later stages (day 7). These results suggest that *Ifng-as1* regulation may become more relevant during sustained Th1 differentiation, rather than at the early stage analyzed in our study.

The distinct effects observed upon deletion of the –70 kb versus +66 kb CBSs on enhancer-promoter interactions are particularly intriguing. We hypothesize that these differences may result from several established CTCF-mediated mechanisms. First, intrinsic differences in CTCF binding affinity at these sites, driven by subtle sequence variations, could influence their ability to stabilize chromatin loops (55, 56). Second, the orientation of CTCF motifs is a well-known determinant of the directionality of loop formation (24, 42); thus, distinct motif orientations at the –70 kb and +66 kb CBSs may impose differential spatial constraints on the *Ifng* locus. Third, the recruitment of specific cofactors to each CBS could further diversify their functional output, leading to distinct impacts on enhancer-promoter communication (57, 58).

In addition to these site-specific effects, large-scale chromatin topology at the *Ifng* locus appears to be evolutionarily conserved. Recent Hi-C analyses demonstrated that both mouse and human CD4^+^ T cells share similar domain organization despite species-specific differences in gene content (13). In particular, human cells harbor an *IL22* TAD anchored between *MDM1* and *IL26*, a cytokine gene absent in mice, yet the overall insulation between the *Il22* and *Ifng* TADs is preserved (13). This cross-species conservation suggests that the higher-order 3D organization of the *Ifng* locus represents a fundamental regulatory mechanism, ensuring appropriate separation of type 1 and type 3 cytokine responses across mammals.

Future studies should aim to dissect these mechanisms, for example by analyzing CTCF binding kinetics, motif orientation relative to interacting elements, and the composition of associated protein interactomes at each CBS.

### Importance of the *Ifng* –70 kb CBS as a TAD boundary and insulator

Loss of CTCF occupancy at TAD boundaries can compromise insulation between chromatin domains, resulting in aberrant enhancer–promoter interactions, ectopic gene activation, and diseases (22, 27, 28). For instance, deletion of a boundary at the *WNT6-EPHA4-PAX3* locus disrupted developmental patterning in mouse limb by enabling inappropriate enhancer activity (28). Similarly, in the mouse α-globin gene clusters, loss of a CBS that partitions the domain into smaller intra-TADs led to dysregulated expression of normally silenced genes (59). At the *Ifng* locus, deletion of the –70 kb CBS led to merging of the *Il22* and *Ifng* TADs in Th1 cells, resulting in dysregulated cytokine expression (13). Furthermore, the *Mdm1* locus, located in the upstream *Il22* TAD, aberrantly interacted with the *Ifng* –30 kb accessible region upon deletion of the boundary at –70 kb, indicating a loss of TAD insulation. These results emphasize the pivotal role of the –70 kb CBS in preventing inappropriate chromatin looping between Th1- and Th17-associated loci. Thus, the –70 kb CBS not only supports proper enhancer–promoter interactions within the *Ifng* TAD but also functions as a robust insulator that restricts cohesin-mediated loop extrusion and prevents ectopic interactions across lineage-specific gene domains.

In our dataset, however, we did not observe significant changes in *Il22* expression, whereas Liu et al. reported its up-regulation (13). The *Iltifb* gene is annotated as a pseudogene, and no detectable expression was found in our analysis. With respect to *Mdm1*, Liu et al. reported no significant changes in its expression, and we did not specifically analyze this gene in our study. These differences highlight potential context-dependent variation in transcriptional regulation at the *Ifng* locus and underscore the complexity of gene regulation within this chromatin domain.

Thus, the –70 kb CBS not only supports proper enhancer–promoter interactions within the *Ifng* TAD but also functions as a robust insulator that restricts cohesin-mediated loop extrusion and prevents ectopic interactions across lineage-specific gene domains. Based on current models, CTCF binding at this site may provide a directional barrier or anchor for cohesin, thereby facilitating loop extrusion and stabilizing enhancer–promoter communication. Although our results support this model, we did not directly assess cohesin occupancy at the –70 kb CBS. Future studies using cohesin ChIP-seq (e.g., Rad21, Smc1) or acute cohesin depletion approaches will be required to provide direct mechanistic evidence.

Interestingly, despite the loss of CTCF binding at the –70 kb or +66 kb CBSs, we did not detect appreciable changes in CTCF occupancy at the other CBSs across the *Ifng* locus. This finding suggests that these sites function as discrete and highly localized architectural elements, with minimal compensatory redistribution of CTCF binding. While this may explain the striking specificity of the transcriptional changes observed by RNA-seq, it does not exclude the possibility of more subtle compensatory mechanisms. Further studies, including genome-wide profiling of CTCF occupancy and chromatin interactions in these mutants, will be required to determine whether other sites adaptively modulate their function to maintain higher-order chromatin organization.

### Functional consequences of *Ifng* –70 kb CBS deletion on host defense and tumor surveillance

IFN-γ production by Th1 and CD8^+^ T cells is critical for eliminating intracellular pathogens and tumors. ΔCBS–70 mice were more susceptible to *C. neoformans* infection, consistent with prior studies showing increased vulnerability to *Toxoplasma gondii* infection upon *Ifng* TAD boundary deletion (13). These results demonstrate that even partial reductions in IFN-γ expression, especially during early Th1 differentiation, can impair host resistance to intracellular pathogens, despite minimal changes to the global transcriptome. Furthermore, ΔCBS–70 mice exhibited reduced tumor rejection of B16 melanoma cells. B16 melanomas are recognized targets for multiple immune effector cell types, and their *in vivo* growth can be eliminated by natural killer cell-mediated cytotoxicity (60), activated macrophages (61), or CD8^+^ T cells (62). Although CD8^+^ T cells are key mediators of anti-tumor immunity, CD4^+^ T cells also make a substantial contribution, as B16 tumors can still be rejected in CD8^+^ T cell-depleted mice (62). Thus, IFN-γ produced by both Th1 and CD8^+^ T cells is essential for robust anti-tumor responses, and reduced IFN-γ production in ΔCBS–70 mice likely underlies their diminished tumor surveillance.

In contrast, ΔCBS–70 mice showed comparable disease severity to WT mice in a T cell transfer colitis model. This is consistent with previous findings that this model is largely driven by the IL-23-Th17 pathway rather than IFN-γ-dependent Th1 responses. Indeed, IL-23 is essential for disease induction, whereas IL-12 is dispensable (63). Thus, the absence of a phenotype in colitis underscores the context-specific requirement for IFN-γ, which is critical in infection and tumor models but less dominant in TH17-driven intestinal inflammation.

### Future perspectives

Finally, although our study focused on the structural role of CTCF at TAD boundaries, it is worth noting that recent work has begun to elucidate determinants of cell type-specific CTCF binding, including chromatin accessibility and sequence context (64). These findings highlight an important future direction for understanding how general architectural proteins achieve lineage-specific regulatory outcomes.

## Conclusion

Our study demonstrates that deletion of the *Ifng* –70 kb CBS at a key TAD boundary disrupts chromatin topology, reduces *Ifng* gene expression, and impairs host defense and tumor surveillance. These findings underscore the essential role of specific CTCF-bound elements in establishing and maintaining enhancer–promoter communication and robust immune function. Furthermore, our results highlight a hierarchical chromatin organization, in which TAD boundaries create a permissive environment for intra-domain regulation, possibly influenced by RNA-mediated modulation. These insights advance our understanding of how chromatin architecture integrates with immune gene regulation and illustrate how noncoding genomic elements can influence disease-relevant immune responses.

## Data Availability

The datasets presented in this study can be found in online repositories. The names of the repository/repositories and accession number(s) can be found in the article/**Supplementary Material**.
