# Supplementary material for: Essential roles of CTCF binding sites at TAD boundaries in modulating chromatin interactions and transcriptional regulation at the Ifng locus
Source: Front Immunol. 2025 Sep 26;16:1667851. doi: 10.3389/fimmu.2025.1667851 (PMC12511032; doi:10.3389/fimmu.2025.1667851)
Supplement: Supplementary file 1 [file DataSheet1.pdf]

frontiers

Supplementary Material

Supplementary Figure 1

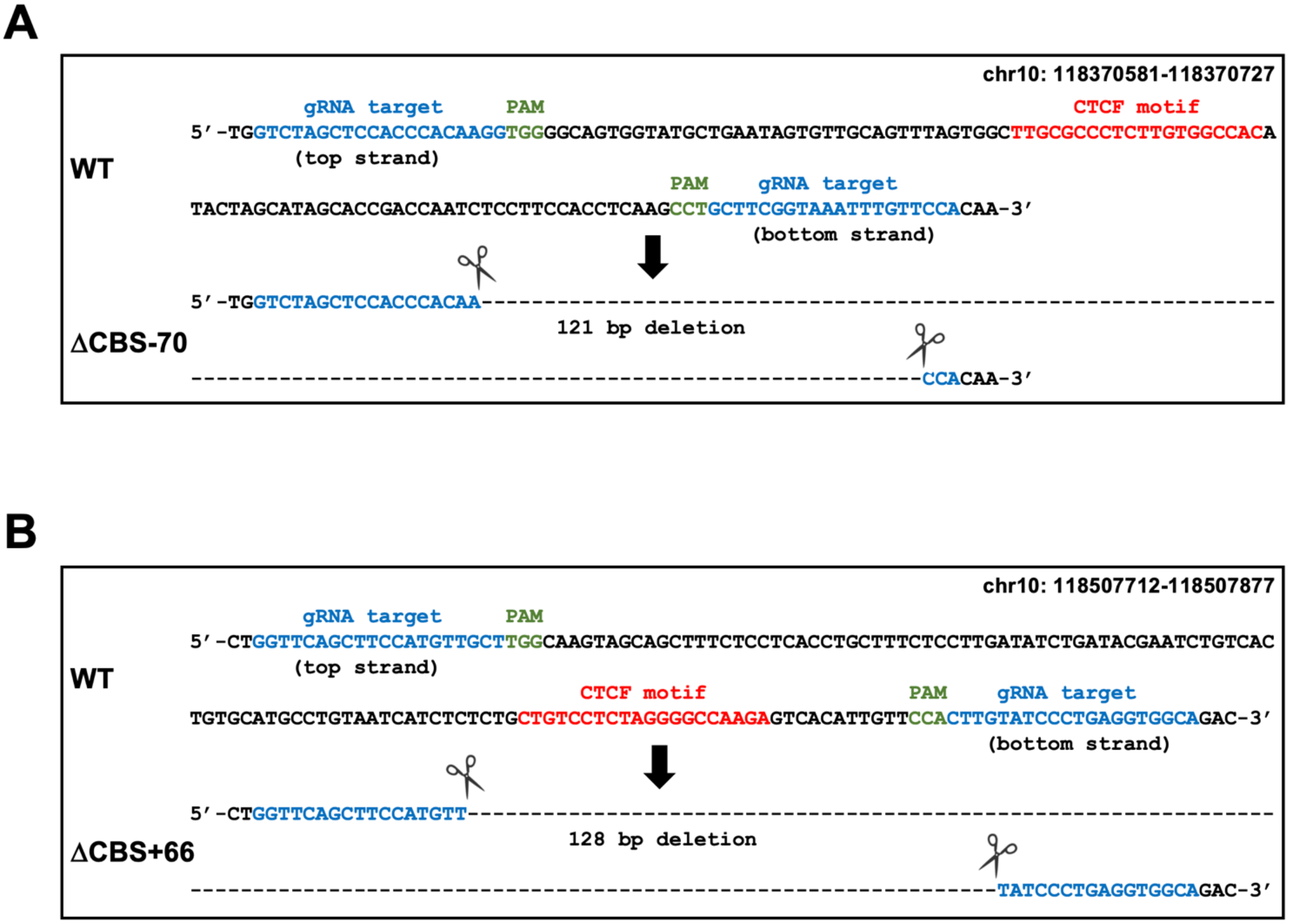

Supplementary Figure 1. Genome editing strategy overview. (A, B) Schematic diagrams of CRISPR-Cas9-mediated deletion of the *Ifng* –70 kb (A) and +66 kb (B) CBSs at the *Ifng* locus. CTCF motifs are shown in red, PAM sequences in green, and gRNAs in blue.

Supplementary Figure 2

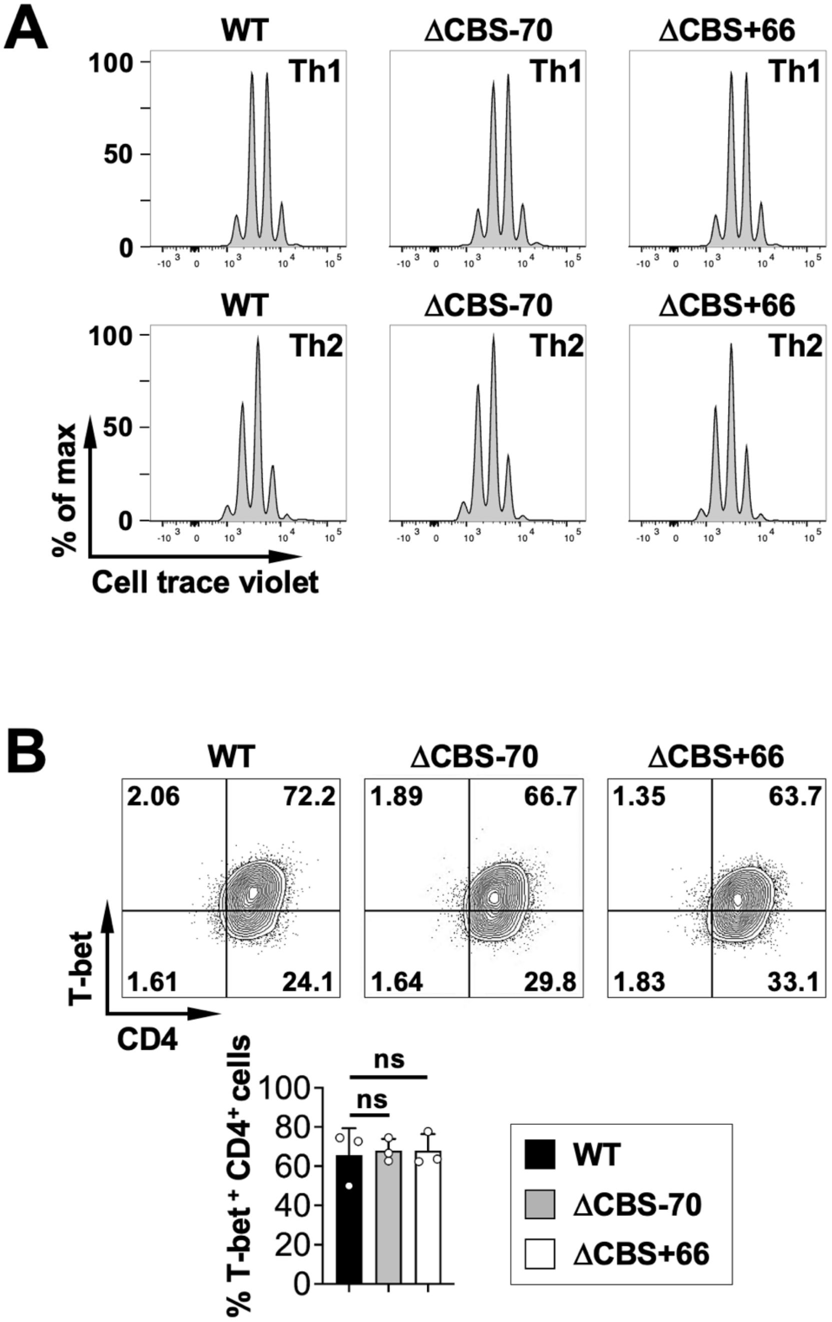

Supplementary Figure 2. Deletion of *Ifng* CBSs does not affect T cell proliferation and T-bet expression. (A) Flow cytometric analysis of cell proliferation. Naive T cells from WT, ΔCBS–70, and ΔCBS+66 mice were FACS-sorted and cultured under Th1 or Th2 (A) or Th1 only (B) polarization conditions for 3 days. (B) T-bet expression was assessed by flow cytometry. Data are representative of three independent experiments. Bar graphs show mean ± SD. Significance: non-paired two-tailed Student's t test. *$p < 0.05$; **$p < 0.01$; ***$p < 0.001$; ns, not significant.
